# Trust Your Gut: Recognizing Whipple’s Disease Beyond the Intestine

**DOI:** 10.7759/cureus.102877

**Published:** 2026-02-03

**Authors:** Rosélia Lima, Maria Soares, Mariana Baptista, Andreia Seixas, Margarida Mota

**Affiliations:** 1 Internal Medicine, Gaia Espinho Local Health Unit, Vila Nova de Gaia, PRT

**Keywords:** case series, constrictive pericarditis, diarrhea, malabsorption, whipple’s disease

## Abstract

Whipple’s disease is a rare, chronic, multisystemic infection caused by *Tropheryma whipplei (T. whipplei)*, classically presenting with gastrointestinal manifestations but frequently involving extraintestinal organs that may delay diagnosis. We report two cases illustrating the clinical spectrum of Whipple’s disease: a 59-year-old man with malabsorption, chronic diarrhea, and weight loss, and a 44-year-old man with inflammatory polyarthralgia and constrictive pericarditis who developed gastrointestinal symptoms eight years later. In both cases, diagnosis was established by duodenal biopsy demonstrating periodic acid-Schiff (PAS)-positive macrophages and polymerase chain reaction confirmation of *T. whipplei*. Both patients were treated with intravenous ceftriaxone followed by long-term trimethoprim-sulfamethoxazole, with favorable clinical outcomes. These cases highlight the diagnostic challenges posed by atypical Whipple’s disease and underscore the importance of considering this diagnosis in patients with unexplained multisystem inflammatory syndromes.

## Introduction

Whipple’s disease is a rare, chronic, multisystemic infection caused by the Gram-positive actinomycete *Tropheryma whipplei (T. whipplei) *[1]. First described in 1907, it predominantly affects middle-aged men of European descent, with an estimated incidence of less than one per million population per year [2]. The small intestine is the primary site of involvement, where infiltration by infected macrophages leads to malabsorption, diarrhea, steatorrhea, and weight loss- the classic presentation [3]. However, impaired bacterial clearance and systemic dissemination through lymphatic and hematogenous pathways may result in extraintestinal involvement, affecting joints, lymph nodes, the heart, and the central nervous system. Such manifestations may precede gastrointestinal symptoms by years, delaying diagnosis [4-6].

In patients with gastrointestinal symptoms, diagnosis is confirmed by upper endoscopy with small bowel biopsies. Histology typically demonstrates periodic acid-Schiff (PAS)-positive macrophages, which are supportive but not entirely specific; therefore, molecular confirmation by polymerase chain reaction (PCR) is recommended. Immunohistochemistry may also support the diagnosis. When results are inconclusive or gastrointestinal symptoms are absent, specimens should be obtained from other sites of involvement, such as synovial fluid, lymph nodes, cerebrospinal fluid, or resected cardiac valves. Noninvasive PCR testing in stool, saliva, or urine has limited sensitivity (approximately 30-60%) and may detect asymptomatic colonization rather than true systemic infection [7,8]. Early recognition is essential, as untreated disease is typically fatal; however, most patients respond favorably to prolonged antibiotic therapy [9].

We present two cases illustrating the clinical spectrum of Whipple’s disease: one with classic gastrointestinal involvement and another with an atypical extraintestinal presentation. The educational purpose of these cases is to emphasize the diagnostic challenges and highlight the need for clinical vigilance in patients with unexplained multisystemic illness.

## Case presentation

Case one - classical presentation

**Figure 1:**
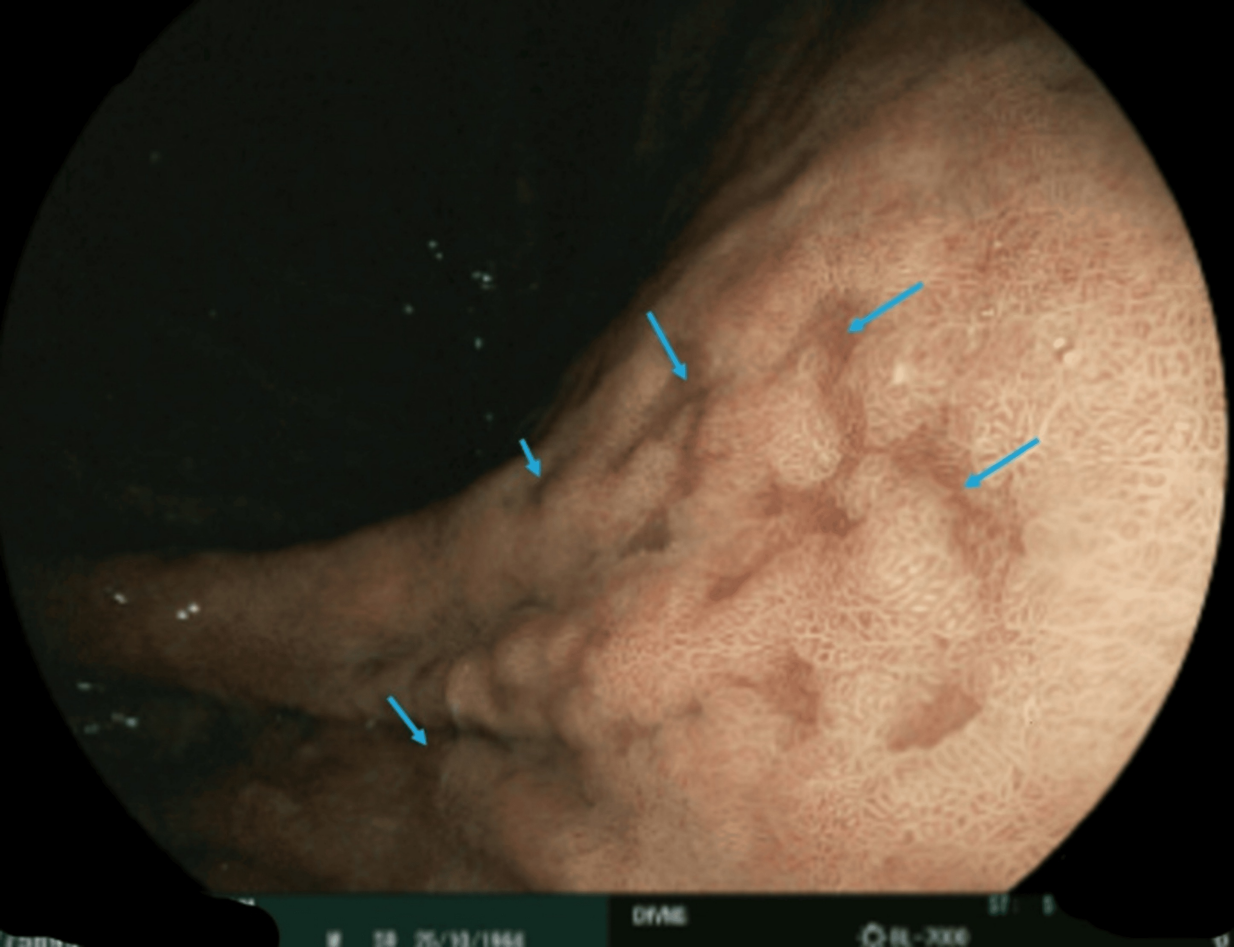
Upper endoscopy showing multiple duodenal lymphangiectasias (arrows), consistent with intestinal involvement in Whipple’s disease.

A 59-year-old man with a history of smoking and chronic alcohol use was admitted with progressive fatigue, unintentional weight loss of 15 kg, and intermittent diarrhea over six months. During diarrheal episodes, he reported 10-15 watery bowel movements per day without blood or mucus. Abdominal pain occurred only before defecation and resolved afterward. He denied fever, nausea, vomiting, anorexia, or night sweats. On presentation, the patient appeared emaciated and dehydrated, with diffuse abdominal tenderness. His weight was 45 kg. Laboratory evaluation confirmed severe malabsorption, with iron- and folate-deficiency anemia (hemoglobin 8.7 g/dL), profound hypoalbuminemia (1.9 g/dL), hyponatremia (128 mmol/L), and hypokalemia (2.8 mmol/L). Abdominal ultrasound was unremarkable, but chest computed tomography (CT) revealed axillary and mesenteric lymphadenopathy. Serologic testing for human immunodeficiency virus and viral hepatitis was negative. Cytomegalovirus infection was excluded through negative blood serology and immunohistochemistry on biopsy specimens, while intestinal tuberculosis was ruled out by negative Ziehl-Neelsen staining, PCR, and mycobacterial cultures from intestinal biopsies. Gastrointestinal malignancy was also considered, but no evidence of cancer was found on Abdominal CT or endoscopic evaluation (upper and lower). Upper endoscopy demonstrated multiple duodenal lymphangiectasias (Figure 1). Duodenal biopsies showed PAS-positive macrophages, and PCR confirmed *T. whipplei*, establishing the diagnosis of Whipple’s disease. The patient was treated with intravenous ceftriaxone for 14 days, followed by oral trimethoprim-sulfamethoxazole planned for 12 months. One month later, he was readmitted with fever and recurrent diarrhea. Repeat duodenal biopsy and PCR for *T. whipplei* were negative, and symptoms resolved with supportive care, consistent with immune reconstitution inflammatory syndrome (IRIS) rather than relapse. Over the course of therapy, he experienced complete resolution of diarrhea, weight gain to 54 kg, and normalization of laboratory abnormalities (hemoglobin 13.4 g/dL, albumin 4.7 g/dL, sodium 141 mmol/L, potassium 4.1 mmol/L).

Case two - atypical presentation

**Figure 2:**
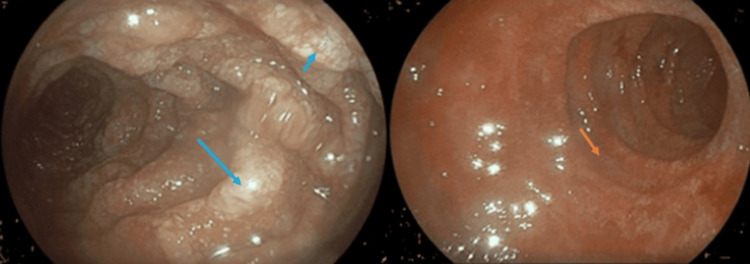
Upper endoscopy of patient two, illustrating alternating whitish edematous areas (blue arrows) with hyperemia and villous flattening in the duodenum (orange arrow), also suggestive of Whipple's disease.

**Figure 3:**
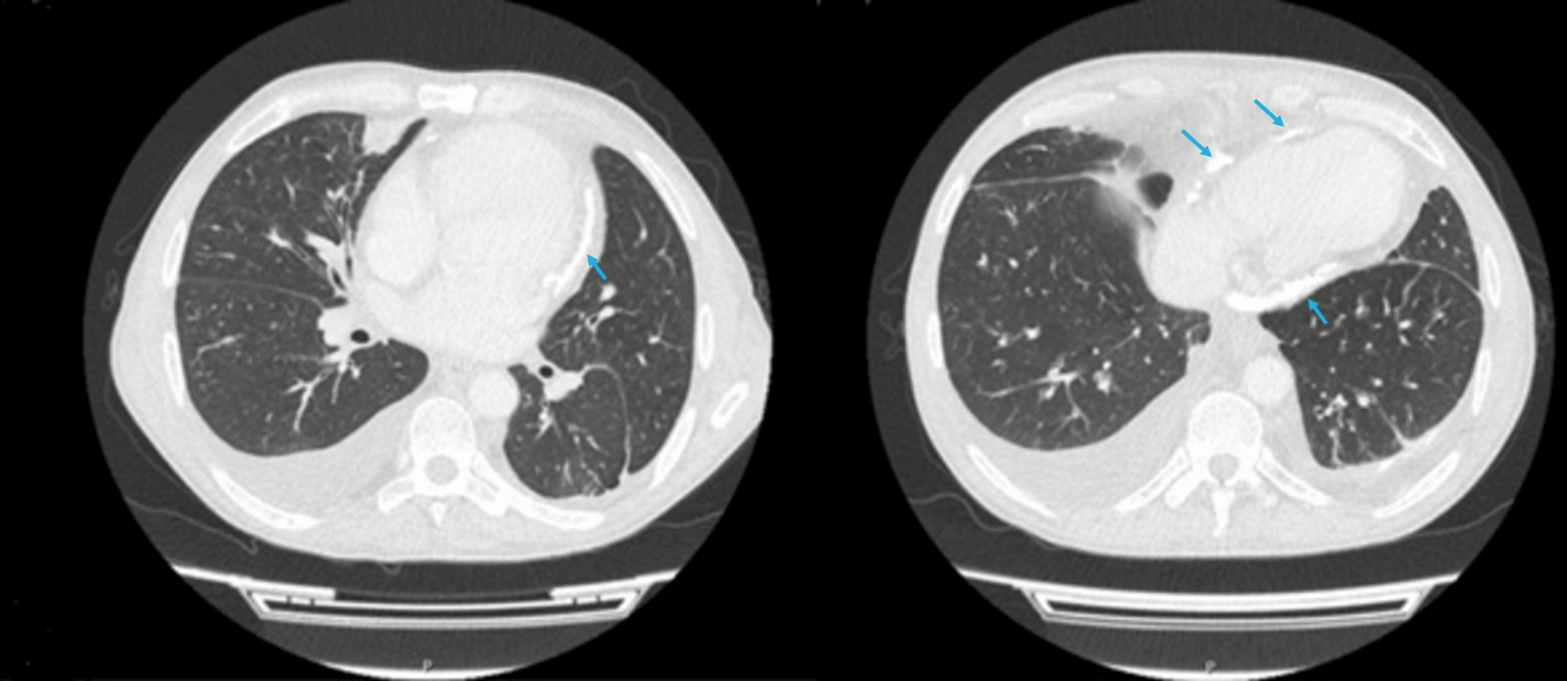
Chest CT scan of patient two demonstrating extensive pericardial calcifications (blue arrows), in particular in the anterior and inferior walls of the pericardium.

A 44-year-old man with no significant past medical history was diagnosed with constrictive pericarditis of unknown etiology. During the initial evaluation, he also reported inflammatory arthralgia affecting both hands and wrists. Laboratory testing demonstrated an elevated erythrocyte sedimentation rate (ESR) of 61 mm/h and a positive antineutrophil cytoplasmic antibodies (ANCA) titer of 1:640, with negative myeloperoxidase (MPO) and proteinase 3 (PR3) antibodies. Chest CT revealed pleural thickening, and articular ultrasound and magnetic resonance imaging of the hands and wrists showed synovitis with synovial fluid accumulation. The autoimmune work-up was unrevealing, with negative antinuclear antibodies, rheumatoid factor, anti-cyclic citrullinated peptide antibodies, and antithyroid antibodies. High-resolution chest CT showed no evidence of systemic vasculitic disease, supporting a nonspecific or false-positive ANCA finding. After eight years of follow-up, the patient developed anorexia, unintentional weight loss of 10 kg, and diarrhea (seven to eight bowel movements per day) with intermittent hematochezia over two months. Inflammatory markers remained elevated ESR (75 mm/h), and anemia developed after the onset of gastrointestinal symptoms (hemoglobin 10.2 g/dL). Upper endoscopy revealed alternating whitish edematous mucosa, hyperemia, and villous flattening (Figure 2). Duodenal biopsies demonstrated PAS-positive macrophages, and PCR confirmed *T. whipplei*. Alternative diagnoses, including fungal infection, Helicobacter pylori, and intestinal tuberculosis, were excluded through negative histopathological and microbiological testing in biopsies. Although central nervous system (CNS) involvement was considered, neurological examination was unremarkable, and lumbar puncture with cerebrospinal fluid analysis was inconclusive. Given the possibility of occult neurological disease, the patient received an extended induction course of intravenous ceftriaxone for four weeks, followed by oral trimethoprim-sulfamethoxazole to complete a 12-month regimen. During treatment, the patient developed peripheral edema. Repeat echocardiography confirmed persistent constrictive physiology, demonstrating pericardial thickening (>3 mm), interventricular septal bounce, and *annulus reversus* on tissue Doppler imaging. Computed tomography further revealed severe circumferential pericardial calcification (Figure 3). Although young, the patient was deemed a poor surgical candidate because of the extensive calcification and operative complexity, making pericardiectomy technically high-risk. Therefore, he was managed conservatively. At four months of follow-up, the patient showed significant improvement, with resolution of polyarthralgia and diarrhea, weight recovery, and progressive normalization of inflammatory markers (ESR 7mm/h).

## Discussion

Whipple’s disease remains frequently misdiagnosed due to its variable and nonspecific clinical presentation. Although it most commonly affects the small intestine, *T. whipplei* can involve nearly any organ system, often mimicking autoimmune, inflammatory, or neoplastic conditions and leading to significant diagnostic delay.

The two cases presented in this manuscript represent opposite ends of the clinical spectrum of Whipple’s disease - one with classic intestinal involvement and another with a prolonged extraintestinal course preceding the onset of gastrointestinal symptoms.

The first case demonstrates the hallmark gastrointestinal features of Whipple’s disease, including chronic diarrhea and malabsorption. In contrast, the second case initially presented with inflammatory polyarthralgia and constrictive pericarditis several years before intestinal manifestations became apparent. Approximately one-third of patients present with atypical forms of Whipple’s disease [10], which can significantly delay diagnosis, particularly when autoimmune serologies such as antineutrophil cytoplasmic antibodies (ANCA) are positive and potentially misleading. In case two, although the ANCA titer was markedly elevated, the absence of MPO/PR3 antibodies and the lack of clinical evidence of vasculitis supported a nonspecific or false-positive finding, contributing to diagnostic uncertainty.

Extraintestinal manifestations such as inflammatory arthralgia, culture-negative cardiac involvement, or unexplained serologic abnormalities may represent early warning signs of Whipple’s disease and can precede gastrointestinal symptoms by many years. Recognition of these features is essential to avoid misdiagnosis as primary rheumatologic or vasculitic disease. Clinicians should therefore suspect Whipple’s disease in patients with unexplained chronic inflammatory polyarthralgia, culture-negative endocarditis or pericarditis, lymphadenopathy, or systemic inflammation, even in the absence of prominent gastrointestinal complaints.

In both patients, the diagnosis was established through duodenal biopsy obtained by upper gastrointestinal endoscopy. However, alternative tissue sampling could also have been considered. In the first case, a lymph node biopsy may have yielded diagnostic material. In contrast, in the second case, analysis of pericardial tissue or synovial fluid could potentially have led to an earlier diagnosis.

Cardiac involvement in Whipple’s disease, although uncommon, is clinically significant and most frequently presents as culture-negative endocarditis or, less commonly, pericarditis. Constrictive pericarditis with pericardial calcification, as observed in our patient, is particularly rare and associated with substantial morbidity. These findings underscore the importance of considering *T. whipplei *infection in cases of unexplained culture-negative cardiac inflammation, especially when accompanied by systemic inflammatory or rheumatologic manifestations [10].

Both patients responded favorably to antibiotic therapy, although one developed IRIS, a recognized transient inflammatory response that may occur during bacterial clearance. Long-term clinical and, when appropriate, molecular follow-up is essential to monitor for relapse, particularly in patients with extraintestinal disease or suspected central nervous system involvement [11].

Overall, these cases highlight the protean nature of Whipple’s disease and reinforce the importance of early recognition, appropriate tissue sampling, and molecular confirmation to ensure timely diagnosis and favorable outcomes. Table 1 provides a comparative summary of the clinical features of both cases.

**Table 1:** Comparison of clinical course of both cases of Whipple's disease. ESR: erythrocyte sedimentation rate; ANCA: antineutrophil cytoplasmic antibodies; PCR: polymerase chain reaction; PAS: periodic acid-Schiff;  IRIS: immune reconstitution inflammatory syndrome.

	Case 1	Case 2
Age at onset	59 years	44 years
Time to diagnosis	6 months	8 years
Gastrointestinal symptoms	Early and prominent	Delayed onset
Extraintestinal involvement	Lymphadenopathy	Constrictive pericarditis; inflammatory arthritis; pleural thickening
Laboratory findings	Anemia (Hb 8.7 g/dL); Hypoalbuminemia (albumin 1.9 g/dL)	Elevated ESR (61 mm/h), Positive ANCA titer (1:640)
Endoscopy findings	Duodenal lymphangiectasia	Edematous whitish mucosa with villous flattening
Histopathology/PCR	PAS-positive macrophages; PCR positive for *T. whipplei*	PAS-positive macrophages; PCR positive for *T. whipplei*
Treatment	Ceftriaxone 2 weeks → Cotrimoxazole 12 months	Ceftriaxone 4 weeks → Cotrimoxazole 12 months
Complications	IRIS	Persistent constrictive pericarditis
Outcome	Symptom resolution; weight gain; laboratory normalization	Symptom resolution; weight recovery; inflammatory marker improvement

## Conclusions

Whipple’s disease should be considered in the differential diagnosis of patients presenting with malabsorption symptoms, arthropathy, or unexplained systemic inflammation, especially when standard investigations are inconclusive. Extraintestinal manifestations may precede gastrointestinal symptoms by years, delaying diagnosis and treatment. Duodenal biopsy with PAS staining and PCR confirmation remains crucial for establishing the diagnosis. Early initiation of appropriate antibiotic therapy results in significant clinical improvement and prevents irreversible organ damage. Awareness of atypical forms-such as pericardial or articular involvement-is vital for timely recognition and management of this potentially fatal yet curable disease.
